# Secondary removal of intramedullary metal debris from a defective Reamer-Irrigator-Aspirator (RIA) reamer head: A case report

**DOI:** 10.1016/j.tcr.2024.101112

**Published:** 2024-09-14

**Authors:** Philipp Vetter, Christian Hübner, Sandro-Michael Heining, Christian Hierholzer, Hans-Christoph Pape

**Affiliations:** Department of Trauma Surgery, University Hospital Zurich, Raemistrasse 100, 8091 Zurich, Switzerland

**Keywords:** Trauma, Bone, Reamer

## Abstract

The Reamer-Irrigator-Aspirator (RIA) device represents a safe and efficient method to harvest autologous bone for grafting. However, hardware failure may occur, for example by breakage of the reamer head with metal debris remaining in the intramedullary canal.

This case report describes the uncomplicated secondary removal of femoral intramedullary metal debris from a broken RIA reamer head; three weeks after the final surgery of a two-stage Masquelet procedure for the treatment of posttraumatic segmental bone loss at the tibia.

## Highlights

• Secondary removal of intramedullary metal debris from Reamer-Irrigator-Aspirator (RIA) breakage was undescribed.
• This case report describes the uneventful course of remaining metal debris.
• And its extraction of from a broken RIA reamer head

## Introduction

The Reamer-Irrigator-Aspirator (RIA) system (DePuy Synthes, Zuchwil, Switzerland) represents an established method for voluminous autologous bone harvesting (25–90 cm^3^) [[1], [2], [3]] by intramedullary reaming of cancellous femoral bone to treat (posttraumatic) bone loss [4], being safe and reliable in use [3]. It can further be used for intramedullary debridement in cases with (concomitant) infection-associated nonunion or low-grade infections [5].

Compared to the most common harvesting site at the iliac crest, it has shown equal to higher union rates, lower complications rates and less donor site complications [[6], [7], [8]].

Despite the overall safe RIA procedure, potential complications include iatrogenic femoral fractures, donor-site morbidity, and hardware-associated complications [9].

Hardware-associated complications occur rarely and comprise reamer head dissociation [4,10] or breakage of the tip of the drive shaft [11]. Their successful management was reported by using a ball-tip guidewire for reamer head extraction [4,10,11].

A case report demonstrated that small metal debris after reamer head breakage was successfully removed intraoperatively using standard instruments [12]. However, there are no reports on secondary removal in the postoperative course.

This case report (general consent was granted) describes the uncomplicated secondary removal of femoral intramedullary metal debris from a broken RIA reamer head, three weeks after the final surgery of a two-stage Masquelet procedure for the treatment of posttraumatic segmental bone loss at the tibia.

## Case presentation

A 43-year-old-male patient who sustained a grade I open and displaced fracture of the left lower leg (AO type 42-B2) following a motorcycle accident. He demonstrated no neurologic deficits and no vascular injuries. In addition, the patient also suffered a fracture of the ipsilateral first and second metatarsal bone.

The patient was initially treated at an outside institution using irrigation, debridement and antegrade tibial nailing.

A subsequent local infection required repeated debridement and irrigation procedures as well as nail removal and application of external fixation with initiation of i.v. antibiotic therapy. A recurrent wound infection led to removal of the external fixation and temporary immobilization using a L-cast application as well as continued antibiotic therapy.

After regression of local signs and blood markers of inflammation, debridement and reaming with microbiologic sampling were performed, followed by insertion of a gentamicine-coated tibial nail (Expert Tibial Nail PROtect (DePuy Synthes, Zuchwil, Switzerland)) and application of a negative wound pressure therapy (NWPT).

The patient was transferred to our hospital for additional treatment. X-rays upon admission revealed that the wedge fragment had dissolved (segmental bone loss of up to 6 cm). Soft tissues surrounding the tibia and the bone defect were debrided, and tissue samples for bacterial analysis were harvested during NPWT exchange.

In the subsequent surgery, there was no sign of infection. A circular gentamicin-loaded cement spacer was implanted for infection control, length restoration and membrane induction (first stage of the Masquelet technique) [13]. The soft tissue defect at the tibia was covered with a thigh flap, while the fractures at the left foot were treated conservatively. No complications occurred.

Three months after spacer implantation, the patient was readmitted for the scheduled second stage of the Masquelet technique.

According to patient preference, treatment of choice was to harvest bone marrow aspirate concentrate (BMAC) by use of a RIA-2 system (DePuy Synthes, Zuchwil), which was inserted into a 3D-printed patient-specific Graft Cage (TRUMATCH, DePuy Synthes, Zuchwil, Switzerland).

The patient was positioned supine. Under sterile conditions, BMAC was harvested with the RIA-2 system in a standard fashion [14].

**Fig. 1:**
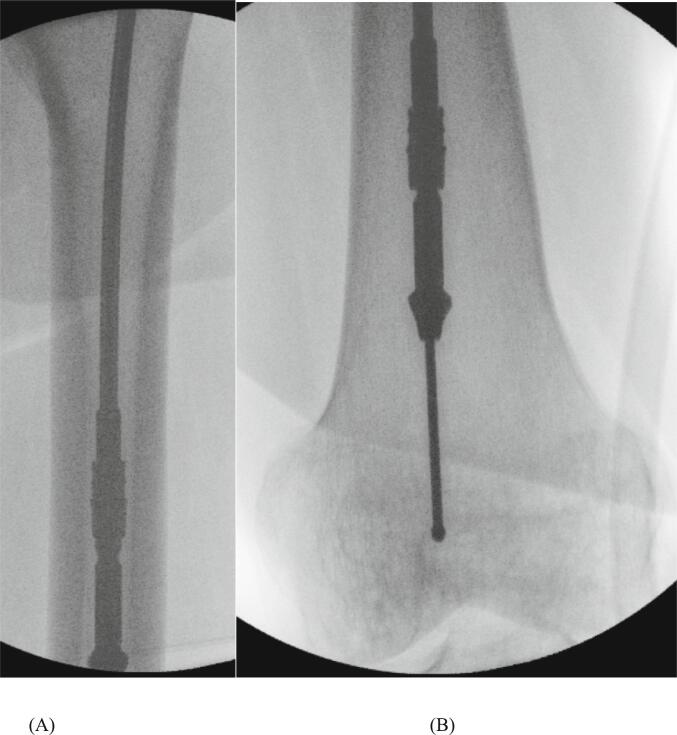
Intraoperative radiographs of reaming at the shaft (A) and at the intercondylar region (B) using the Reamer-Irrigator-Aspirator system.

**Fig. 2:**
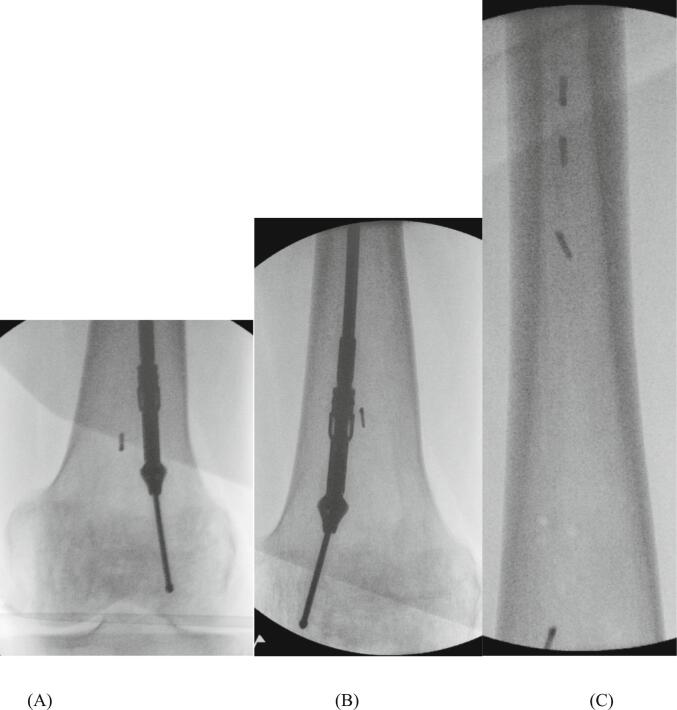
Intraoperative radiographs of small metal debris parts of the broken reamer head in the supracondylar region (A, B), and in the intramedullary canal (C).

Progressive reaming with the correctly assembled system was performed at the femoral shaft and in both condyles (reaming size increasing stepwise from 10 to ultimately 12.5 mm) (Fig. 1), allowing for sufficient harvesting volume. Care was taken to advance the reamer head slowly with low-pressure and high speed. Despite this, drilling appeared suddenly irregular. Fluoroscopy revealed small metal debris parts of the defective reamer head in the intramedullary canal and the intercondylar region (Fig. 2), which could not be removed during the operation. The wound was closed in standard fashion.

On the tibial side, planned treatment with cement spacer removal and implantation of the patient-specific Graft Cage (TRUMATCH, DePuy Synthes, Zuchwil, Switzerland), filled with the RIA-2-harvested BMAC was realized as planned. The wound was closed per standard procedure.

**Fig. 3:**
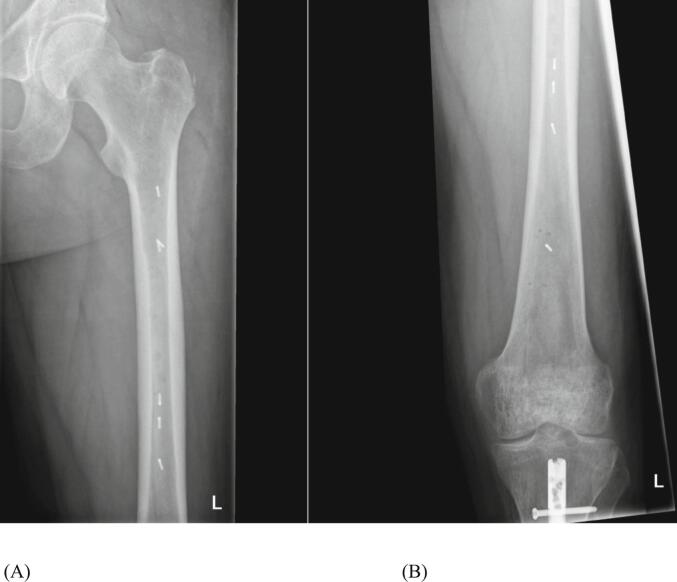
Postoperative radiographs of small metal debris parts of the broken reamer head in the intramedullary canal (A) and in the supracondylar region (B).

**Fig. 4:**
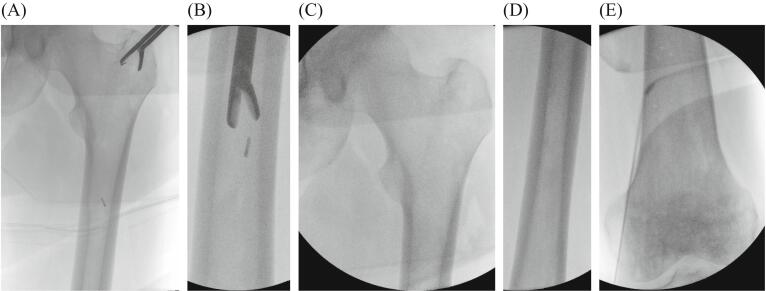
Intraoperative radiographs during the revision surgery, showing fluoroscopic identification (A) and removal of metal debris parts (B) without any residues at the proximal femur (C), shaft (D) or condylar region (E).

**Fig. 5:**
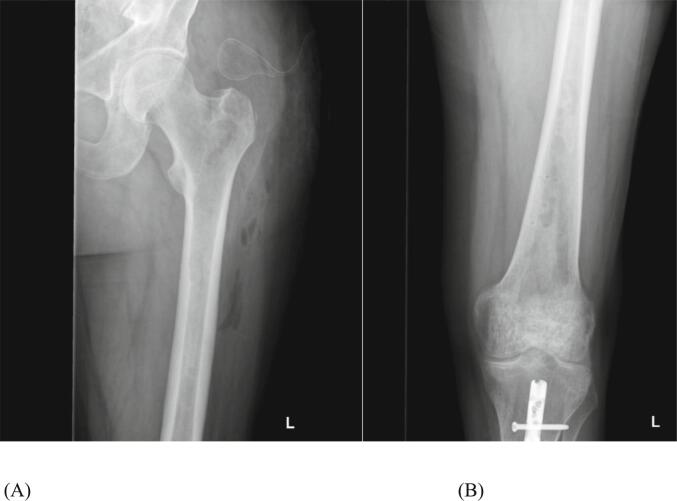
Postoperative radiographs after revision surgery demonstrating complete removal of metal debris at the proximal (A) and distal (B) femur.

Postoperatively, removal of the small metal debris parts of the broken reamer head (Fig. 3) was scheduled for three weeks after the initial reaming, using the same approach at the proximal femur. The postoperative course until revision surgery was medically unremarkable. During revision surgery, the metal parts were identified under fluoroscopy and carefully mobilized with a new reamer head. They were easily removed using a long grasper (Fig. 4, Fig. 5).

The postoperative course after revision surgery was medically uneventful. Osseous consolidation allowed for progressive weight bearing and mobilization of the patient.

## Discussion

The RIA2 system represents a safe and reliable technique for reaming and harvesting of abundant reaming graft material [3]. Hardware-associated complications are rare and comprise dissociation [4,10] or breakage of the reamer head [11]. In these studies of Kanakaris et al. [4], Cipriano et al. [10] and Eisenstein et al. [11], the reamer head was successfully retrieved with a ball-tip guidewire.

No metal debris was found in these studies, which can be ascribed to technical aspects (assembly, dysfunction) or surgical aspects (force of advancing the reamer).

Chloros et al. [12] described the first case of small metal debris after reamer head breakage, which was successfully extracted completely during surgery in a quick and easy manner with standard instruments. A secondary removal of such debris has not been described previously.

In this case presentation, we describe for the first time the painless deposition followed by, several weeks later, secondary removal of small metal debris after reamer head breakage of the RIA-2 system. The case report suggests that immediate removal is not mandatory and may be postponed to a secondary surgery, which may especially be relevant in more extensive and demanding index surgeries for patient and surgeon safety.

Yet, general practical principles should be considered for a successful RIA procedure: Preoperative planning with measuring of the intramedullary diameter (especially the isthmus) is essential. Reaming is initiated with a 10 mm diameter and increased by 0.5 mm, according to the intramedullary diameter. The reamer should be advanced with manual low-pressure at high speed under fluoroscopy to avoid iatrogenic penetration of the femoral cortex. Using an oversized reamer head causes excessive stress at the cortex, which can cause breakage of the wings of the reamer head. Here, manual over-bending of the ball-tip guidewire is to be avoided as the reamer head may get stuck at this point. Drilling at the condylar region should be performed centrally, then laterally and medially to be able to harvest sufficient cancellous bone. After reaming, the reamer should be removed slowly and irrigation must be paused to avoid substantial aspiration which can lead to a notable blood loss.

In addition to bone harvesting, the RIA system can also be used for intramedullary debridement in cases with infection-associated nonunion or low-grade infections [5] (with exchange of intramedullary nailing) to debride the canal of infectious burden and to reduce the risk of dissemination into soft tissue or systemic circulation [5]. In these situations, RIA demonstrated greater efficacy in collection of infected medullary bone tissue compared to conventional reaming [15] and enabled the detection of additional relevant pathogens compared to standard tissue samples [16]. Its use in posttraumatic infections was associated with better functional outcomes [17].

Despite the uncomplicated course since the initial reaming, including the revision surgery, overall surgical time was longer and utilization of removal instruments was required.

These findings emphasize the relevance of the above-mentioned general principles in RIA procedures for optimal treatment.

## Conclusion

Presence of small metal debris after reamer head breakage of the RIA2 system is rare. The surgical effort to remove metal debris may be extensive and may prompt the surgeon to leave it in place.

The current case report shows that secondary removal is feasible with standard instruments and is not associated with a complicated course after RIA-based femoral bone harvesting.

Following bone grafting for posttraumatic segmental bone loss and revision surgery, the patient progressed and rehabilitation therapy was performed as planned.

## Consent for publication

General Consent was granted by the described patient.

## Funding

This research did not receive any specific grant from funding agencies in the public, commercial, or not-for-profit sectors.

## CRediT authorship contribution statement

**Philipp Vetter:** Data curation, Formal analysis, Investigation, Software, Visualization, Writing – original draft. **Christian Hübner:** Project administration, Writing - review & editing. **Sandro-Michael Heining:** Project administration, Writing – review & editing. **Christian Hierholzer:** Project administration, Writing – review & editing. **Hans-Christoph Pape:** Conceptualization, Investigation, Methodology, Project administration, Resources, Supervision, Validation, Writing – review & editing.

## Declaration of competing interest

None.

## Data Availability

Production data is available upon reasonable request.
